# Therapeutic advances in leiomyosarcoma

**DOI:** 10.3389/fonc.2023.1149106

**Published:** 2023-03-08

**Authors:** Kristine Lacuna, Sminu Bose, Matthew Ingham, Gary Schwartz

**Affiliations:** Division of Hematology and Medical Oncology, Department of Medicine, Columbia University Irving Medical Center, New York, NY, United States

**Keywords:** sarcoma, soft tissue sarcoma (STS), leiomyosarcoma (LMS), therapeutics, clinical trials

## Abstract

Leiomyosarcoma is an aggressive mesenchymal malignancy and represents one of the most common subtypes of soft tissue sarcomas. It is characterized by significant disease heterogeneity with variable sites of origin and diverse genomic profiles. As a result, the treatment of advanced leiomyosarcoma is challenging. First-line therapy for metastatic and/or unresectable leiomyosarcoma includes anthracycline or gemcitabine based regimens, which provide a median progression-free survival time of about 5 months and overall survival time between 14-16 months. Effective later-line therapies are limited. Molecular profiling has enhanced our knowledge of the pathophysiology driving leiomyosarcoma, providing potential targets for treatment. In this review, we explore recent advances in our understanding of leiomyosarcoma tumor biology and implications for novel therapeutics. We describe the development of clinical trials based on such findings and discuss available published results. To date, the most promising approaches for advanced leiomyosarcoma include targeting DNA damage repair pathways and aberrant metabolism associated with oncogenesis, as well as novel chemotherapy combinations. This review highlights the recent progress made in the treatment of advanced leiomyosarcoma. Ongoing progress is contingent upon further development of clinical trials based on molecular findings, with careful consideration for clinical trial design, strong academic collaborations, and prospective correlative analyses.

## 1 Introduction

Leiomyosarcoma (LMS) is a malignant neoplasm of smooth muscle differentiation and is one of the most common subtypes of soft tissue sarcomas (STS) in adults, representing 10-20% of new diagnoses (1, 2). LMS is itself a heterogeneous disease with variable sites of origin, clinical course, and response to therapy, making the treatment of LMS challenging. Common anatomical sites include the uterus, abdomen, retroperitoneum, and larger blood vessels. LMS of the extremity is less common, accounting for 10-15% of limb sarcomas, with predilection for the thigh (3). Cure may be achieved in patients with localized LMS who undergo surgery, however 40% of cases will still develop local recurrence and/or metastatic disease, most commonly to lung (4). Patients with advanced LMS are typically treated with chemotherapy, either with gemcitabine or doxorubicin based regimens in the first-line setting. Beyond first-line chemotherapy, which provides a median progression-free survival (PFS) of only about 5 months, there are limited treatment options for advanced disease (5).

Molecular profiling has aided in understanding the biology of LMS, providing implications for novel targeted therapies. Based on such profiles, approaches to LMS have evolved and are currently being explored in ongoing clinical trials. In this review article, we describe recent advances in the treatment of advanced LMS. A subset of ongoing clinical trials for patients with LMS is highlighted in **Table 1**.

**Table 1:** Selected available systemic therapies for advanced leiomyosarcoma.

Regimen	Line of therapy	First Author	Phase	Type of Sarcoma	PFS (months)	RR (%)	OS (months)
Doxorubicin v gemcitabine plus docetaxel	First	Seddon (5)	3	STS	5.3 v 5.5	19 v 20	16.3 v 14.5
Trabectedin v dacarbazine Trabectedin v dacarbazine: uLMS subgroup analysis	> 1	Demetri (6) Hensley (7)	3	STS uLMS	4.2 v 1.5 4.0 v 1.5	9.9 v 6.9 11 v 9	12.4 v 12.9 13.4 v 12.9
Pazopanib v placebo Pazopanib: uSTS v non-uSTS subgroup analysis	> 1	Van der Graaf (8) Benson (9)	3	STS STS	4.6 v 1.6 3.0 v 4.5	6 v 0 11.4 v 10.7	12.5 v 10.7 17.5 v 11.1
Eribulin v dacarbazine Eribulin v dacarbazine: LMS subgroup analysis	> 1	Schoffski (10) Blay (11)	3	LMS+LPS LMS	2.6 v 2.6 2.2 v 2.6	4 v 5 5 v 7	13.5 v 11.5 12.7 v 13

LMS, leiomyosarcoma; uLMS, uterine leiomyosarcoma; STS, soft tissue sarcoma; uSTS, uterine soft tissue sarcoma; LPS, liposarcoma; RR, response rate; PFS, progression free survival; OS, overall survival; v, versus.

## 2 Tumor biology

The pathophysiology of LMS is complex, making the discovery of effective and targeted treatments challenging. LMS lacks a defining genomic alteration and is instead characterized by substantial mutational heterogeneity with frequent whole-genome duplication, widespread DNA copy-number alterations, and chromothripsis (12–15). The most consistent genomic alterations seen across several studies include mutations or deletions in the tumor suppressors *RB1, PTEN*, and *TP53*. Targetable, activating mutations in oncogenes are rare. Molecular profiling has also uncovered recurrent alterations in telomere maintenance genes such as *ATRX* and homologous recombination DNA repair genes. There has also been evidence for immune infiltration in LMS (16, 17); however, tumor mutational burden is low and microsattelite instability is rare (18, 19).

Due to this genomic heterogeneity, multiomic molecular profiling studies have attempted to further categorize subtypes within LMS. Because LMS may be found in several anatomical sites, investigators asked whether different sites represent molecularly distinct diseases, in particular uterine LMS versus nonuterine/soft-tissue LMS. This is of importance as many clinical trials for LMS are designed to include all anatomical subtypes. From an analysis of 1115 LMS tumors, results suggest that uterine LMS represents a molecularly distinct disease with varying genomic alterations compared with nonuterine LMS (17).

Other studies have identified LMS subtypes that do not necessarily reflect anatomical sites of origin. Molecular subtypes associated with distinct clinical outcomes have been identified by several studies (16, 20–23). Dr. Guo and colleagues demonstrated three reproducible molecular subtypes: Subtype I expressed genes associated with smooth muscle differentiation and demonstrated favorable outcome versus subtype II which expressed less smooth muscle differentiation and had worse prognosis. Subtypes I and II included both uterine and nonuterine LMS whereas subtype III consisted mainly of uterine LMS, which demonstrated intermediate outcome (20). Similarly, Dr. Anderson and colleagues identified three distinct molecular subtypes of LMS that correlate with patient survival. Subtype I (uterine and nonuterine LMS) and subtype III (mainly uterine LMS) harbored a higher overall burden of somatic mutations and were associated with worse survival compared to subtype II (nonuterine LMS of the abdomen/extremity). Furthermore, subtype I was associated with myogenic dedifferentiation and high immune infiltration (16). These data suggest that a subset of uterine LMS behave as an independent molecular subtype while another subset of uterine LMS joins nonuterine LMS to become part of the other identified subtypes.

The identification of varying molecular patterns within LMS highlights the challenges of studying this disease. As of now, patients with LMS are enrolled onto clinical trials as a homogenous entity, ocasionally considering site of disease (uterine versus nonuterine). However, as we have seen, patients with LMS (including those with the same anatomical site) can display vastly different outcomes based on subtype. This can make it difficult to interpret overall results from a trial, as clinically meaningful outcomes may not be directly apparent for certain populations within LMS. Future molecular studies should focus on identifying actionable targets and biomarkers within these LMS subtypes, which may then be incorporated into future clinical designs and subgroup analyses. Enrollment and treatment selection based on molecular data may ultimately reveal a preferential response for an LMS subtype that would not otherwise be identified.

## 3 Approved treatments for advanced LMS

### 3.1 Early line therapies

LMS demonstrates moderate sensitivity to chemotherapy, with uterine LMS being more responsive compared to other anatomical sites (24). In the first-line setting, doxorubicin or gemcitabine based regimens are commonly used. In the phase 2 trial, Gemcitabine and Docetaxel versus Doxorubicin as First-Line Treatment in Previously Untreated Advanced Unresectable or Metastatic Soft-Tissue Sarcoma (GeDDiS), both regimens demonstrated comparable efficacy in STS, including LMS. For gemcitabine and docetaxel versus doxorubicin, there were no significant differences in median progression free survival (PFS) (5.5 versus 5.3 months) or overall survival (OS) (14.5 versus 16.3 months), and objective response rates (ORR) were similar (20% versus 19%). Quality-of-life assessments were compared between the two treatment groups at 12 weeks. There was no significant difference between the two groups at 12 weeks however the mean global health status score was numerically higher in the doxorubicin group versus gemcitabine and docetaxel. This may influence treatment decision for select patients (5).

Although subgroup analysis performed within the GeDDiS trial demonstrated no evidence of differential treatment effect by histologic subtype, other studies in uterine LMS have suggested unique sensitivity to gemcitabine and docetaxel. In a phase 3 study of gemcitabine and docetaxel compared with gemcitabine alone in patients with metastatic soft tissue sarcomas (SARC002), the combination showed superior objective response, PFS and OS. This study also confirmed a higher sensitivity of LMS to gemcitabine and docetaxel compared with other histologic subtypes (25). Subsequently, in a phase 2 study of gemcitabine and docetaxel as first-line treatment for uterine LMS, the ORR was 35.8% with complete response seen in 4.8%, partial response in 31% and stable disease in 26.2% of patients (26). Cross-study comparison is limited however these response findings may imply a more favorable benefit of gemcitabine and docetaxel in uterine LMS versus response data seen in other studies such as GeDDiS. As a result, some prefer gemcitabine and docetaxel as first-line treatment for uterine LMS. Choice of first-line treatment remains individualized, with consideration of many factors including patient preference, performance status, and comorbidities.

Other gemcitabine-based regimens may be considered for early-line treatment of LMS, such as gemcitabine plus vinorelbine and gemcitabine plus dacarbazine. In a phase 2 study of gemcitabine plus vinorelbine in patients with advanced soft tissue sarcomas including LMS who received ≤ 1 prior therapy, clinical benefit (defined as complete response, partial response, or stable disease at > 4 months) was seen in 25% of patients (27). In a randomized phase II study comparing gemcitabine plus dacarbazine versus dacarbazine alone in patients with previously treated STS, median PFS was 4.2 months versus 2 months, median OS was 16.8 months versus 8.2 months, with higher ORR of 49% versus 25% (28). As a result, this regimen may be considered for patients with LMS who failed anthracycline-based treatment.

Early-line therapy for LMS also includes the combination of doxorubicin plus dacarbazine. In a retrospective study of doxorubicin plus dacarbazine, doxorubicin plus ifosfamide or doxorubicin alone as first-line treatment for advanced LMS, 303 patients were included for which 117 (39%) received doxorubicin plus dacarbazine, 71 (23%) received doxorubicin plus ifosfamide, and 115 (38%) received doxorubicin alone. The estimated median PFS was 9.2 months, 8.2 months, 4.8 months, median OS was 36.8 months, 21.9 months, 30.3 months, with ORR of 30.9%, 19.5% and 25.6% for doxorubicin plus dacarbazine, doxorubicin plus ifosfamide, and doxorubicin alone, respectively (29). These data demonstrate favorable activity of doxorubicin plus dacarbazine in LMS and warrant further investigation in prospective clinical trials.

### 3.2 Later line therapies

Later-line treatment of LMS includes trabectedin, pazopanib, and other chemotherapy agents. Trabectedin is approved in patients with advanced liposarcoma (LPS) or LMS who received prior treatment with anthracyclines. In the randomized phase 3 study of trabectedin versus dacarbazine for metastatic LPS or LMS after failure of conventional chemotherapy, trabectedin demonstrated superior median PFS versus dacarbazine (LMS: 4.3 versus 1.6 months). However, there were no significant differences in OS (12.4 versus 12.9 months) or ORR (9.9 versus 6.9%) (6). In a uterine LMS specific subset analysis of this phase 3 trial, trabectedin provided a median PFS of 4.0 months compared with 1.5 months for dacarbazine, with an ORR of 11% (7). From these data, trabectedin was approved for advanced LMS in October 2015.

Pazopanib is another approved treatment for patients with advanced STS who have previously received chemotherapy, with activity in LMS. Pazopanib is a small-molecule tyrosine kinase inhibitor that inhibits vascular endothelial growth factor (VEGF) receptor, platelet-derived growth factor (PDGF) receptor, and c-KIT (30). In the randomized phase 3 study of pazopanib for metastatic STS (PALETTE), pazopanib demonstrated superior PFS versus placebo (4.6 versus 1.6 months). However, there were no differences in OS (12.5 versus 10.7 months) and objective responses occurred in only 6% of patients (8). In a uterine LMS specific subset analysis, pazopanib provided a median PFS of 3.0 months, OS of 17.5 months, and ORR of 11% (9).

Other chemotherapy agents are also considered for later-line treatment of LMS. Although inferior to trabectedin, dacarbazine demonstrates activity in LMS and is used in the later-line setting (6, 10, 11). In a phase 3 trial of eribulin versus dacarbazine in previously treated patients with advanced LPS or LMS, OS was improved in patients assigned to eribulin. However, an LMS-specific subset analysis demonstrated comparable efficacy for eribulin and dacarbazine (10). Eribulin was approved in January 2016 for LPS, but not for LMS, though the drug is sometimes used for later-line treatment of LMS.

Early-line treatment options for LMS provide a median PFS of approximately 5 months with a median OS of 14-16 months. Later-line regimens are less efficacious, with a median PFS of about 3-4 months, median OS of 12-13 months, with low response rates. Results are summarized in **Table 2**. There is an urgent need for improved treatment options for patients with LMS. Based on a greater understanding of LMS tumor biology, novel approaches to LMS have evolved and are currently being explored in ongoing clinical trials.

**Table 2:** Selected ongoing clinical trials in leiomyosarcoma.

NCT Identifier	Title	Phase	Line of Therapy	Eligible Subtypes	Status
NCT05633381	Testing Olaparib and Temozolomide Versus the Usual Treatment for Uterine Leiomyosarcoma After Chemotherapy Has Stopped Working	2/3	> 2	uLMS	Recruiting
NCT05116683	ATX-101 in Advanced Dedifferentiated Liposarcoma and Leiomyosarcoma (ATX-101)	2	> 1	LMS	Recruiting
NCT04807816	Targeting ATR in Soft-tissue Sarcomas (TARSARC)	2	0-4	LMS	Recruiting
NCT03536780	Avelumab in Combination With Gemcitabine in Advanced Leiomyosarcoma as a Second-line Treatment (EAGLES)	2	> 1	LMS	Recruiting
NCT04577014	Retifanlimab (Anti-PD-1 Antibody) With Gemcitabine and Docetaxel in Patients With Advanced Soft Tissue Sarcoma	1/2	First	STS including LMS	Recruiting
NCT03138161	SAINT: Trabectedin, Ipilimumab and Nivolumab as First Line Treatment for Advanced Soft Tissue Sarcoma	1/2	>1 (Phase 1); First (Phase 2)	STS including LMS	Recruiting
NCT04551430	Cabozantinib Combined With PD-1 and CTLA-4 Inhibition in Metastatic Soft Tissue Sarcoma	2	2-3	STS including LMS	Recruiting
NCT04624178	A Study of Rucaparib and Nivolumab in People With Leiomyosarcoma	2	2-4	LMS	Not recruiting*
NCT04242238	A Phase 1b Dose Escalation and Dose Expansion Study of a CSF1R Inhibitor (DCC-3014) Administered Concurrently With an Anti-PD-L1 Antibody (Avelumab) in Patients With Advanced High-grade Sarcoma	1b	> 1	STS including LMS	Not recruiting*
NCT03719430	APX005M and Doxorubicin in Advanced Sarcoma	2	Any	STS including LMS	Recruiting
NCT04996004	A Study to Learn About the Study Medicine (Called TTI-621) Given Alone and in Combination With Doxorubicin in People With Leiomyosarcoma (TTI-621-03)	2	Second	LMS	Recruiting
NCT04200443	Cabozantinib and Temozolomide for the Treatment of Unresectable or Metastatic Leiomyosarcoma or Other Soft Tissue Sarcoma	2	0-5	STS including LMS	Recruiting
NCT03016819	Phase III Trial of Anlotinib, Catequentinib in Advanced Alveolar Soft Part Sarcoma, Leiomyosarcoma, Synovial Sarcoma (APROMISS) (APROMISS)	3	> 1	STS including LMS	Not recruiting*
NCT05269355	A Study of Unesbulin in Participants With Advanced Leiomyosarcoma (LMS) (SUNRISELMS)	2/3	> 1	LMS	Recruiting

LMS, leiomyosarcoma; uLMS, uterine leiomyosarcoma; STS, soft tissue sarcoma.

*Not recruiting at the time of publication.

## 4 Novel approaches to LMS

### 4.1 Targeting DNA repair pathways

Homologous recombination (HR) comprises a series of interrelated pathways that function in the repair of double-stranded DNA breaks (31). HR deficiency is seen in tumors with loss of *BRCA1/2* function as well-described in ovarian, breast, prostate, and pancreatic cancers. More recently, research has been directed at the concept of “BRCAness” which is a condition in which tumors lack mutations in *BRCA1/2* but harbor alterations in other HR pathway genes resulting in HR deficiency (32). Tumors that display “BRCAness” due to defects in the HR DNA repair pathway may offer opportunities for targeted therapy.

Normally, DNA damage repair is a carefully regulated process in which single-stranded DNA breaks are identified by PARP, resulting in the recruitment of other DNA damage response proteins. PARP inhibitors (PARPi) result in trapping of PARP at sites of DNA damage, causing replication fork arrest and lethal double-stranded DNA breaks. To resolve this PARP-DNA interaction, HR repair is needed to accurately fix the resulting double-stranded DNA breaks and restart stalled replication forks. In tumors that are HR deficient, double-stranded break repair is imprecise leading to DNA damage accumulation, progressive genomic instability, and cell death (33, 34). Patients with HR deficient tumors may respond more efficaciously to PARPi-based treatment strategies.

LMS, particularly uterine LMS, harbors frequent defects in DNA damage repair based on research from several groups (12, 13, 16, 32, 35–38). In whole-exome and transcriptomic sequencing of 49 LMS patients, deleterious alterations in HR genes were found in the majority of tumors. Enrichment of a mutational signature associated with defective HR repair (Alexandrov-COSMIC mutational signature AC3) was found in at least 57% of cases. In clonogenic assays, LMS cell lines harbored multiple alterations in HR genes and were responsive to the PARPi olaparib in a dose-dependent fashion (13). In a separate cohort of 170 LMS patients from The Ohio State University and the Cancer Genome Atlas, deleterious HR pathway alterations were identified in 23% of patients with uterine LMS and 15% with nonuterine LMS. *BRCA1/2* loss was seen in 10% of the uterine LMS cases and 1% of nonuterine LMS cases. Four uterine LMS patients were treated with off-label olaparib and demonstrated evidence of clinical benefit (35). In another analysis of 211 LMS cases from Memorial Sloan Kettering Cancer Center, deleterious alterations in HR pathway genes were highlighted in uterine LMS compared with nonuterine LMS. About 18% of patients with uterine LMS harbored an HR pathway alteration versus 10% seen in nonuterine LMS (36, 37). Lastly, in a pan-cancer analysis of germline and somatic *BRCA* alterations of several cancers, uterine LMS harbored the highest rate of somatic homozygous *BRCA2* deletion (38).

To investigate the “BRCAness” of LMS and potential for novel targeted therapy, further preclinical evaluations of PARPi have been performed. In the Schwartz laboratory at Columbia University, *in vitro* studies demonstrated limited activity of PARPi monotherapy with olaparib in LMS cell lines. As a result, combination therapies were investigated to potentiate the effects of PARPi. Anti-neoplastic agents such as temozolomide and trabectedin induce DNA damage and are thought to potentiate PARP trapping, leading to increased apoptosis. Additional *in vitro* studies supported this hypothesis in which concurrent treatment with olaparib plus a DNA damaging agent (temozolomide) provided a profound reduction in cell viability of ≥ 90% (32).

PARPi combinations are now being investigated in prospective clinical trials for LMS. The combination of olaparib plus trabectedin was studied in a phase 1b trial by Dr. Grignani and colleagues, where this combination was deemed safe and well-tolerated, with a recommended phase 2 dose (RP2D) of trabectedin at 1.1 mg/m^2^ every 3 weeks plus olaparib 150 mg twice a day (39). This led to a phase 2 study of trabectedin in combination with olaparib for advanced unresectable or metastatic sarcoma, which included a cohort of patients with LPS and LMS. Results were presented at the Connective Tissue Oncology Society (CTOS) Annual Meeting in 2022 which demonstrated significant toxicity with this regimen, resulting in frequent dose delays/modifications and discontinuation in 19% of patients overall. For the LMS/LPS cohort, the median PFS was 3.5 months. There were no confirmed objective responses, with best overall response of stable disease in 75% and progressive disease in 25% of patients. As a result, enrollment to stage 2 for the LPS/LMS cohort was not opened (40). A phase 2 study of temozolomide and olaparib for advanced uterine LMS provided encouraging results. In this trial by Dr. Ingham and colleagues, 22 patients who received a median of three prior lines of therapy were treated with temozolomide 75 mg/m^2^ once daily in combination with olaparib 200 mg twice daily on days 1-7 of 21-day cycles. The temozolomide plus olaparib combination provided a median PFS of 6.9 months and an ORR of 27%, with a median duration of response of 12 months. Hematologic toxicity was common, as 77% of patients experienced grade 3/4 neutropenia and 32% of patients experienced grade 3/4 thrombocytopenia; however, this toxicity was manageable with dose reduction and there were no events of neutropenic fever or bleeding (41). In correlative analysis, alterations in HR genes including *PALB2* and *RAD51B* or absence of *RAD51* foci formation by a functional assay were observed in patients with prolonged PFS (42). A randomized phase 2/3 trial of olaparib plus temozolomide versus investigator’s choice for uterine LMS after chemotherapy failure has initiated recruitment (NCT05633381).

Another potential therapy targeting DNA damage repair pathways in LMS includes the cell-penetrating peptide, ATX-101. Proliferating cell nuclear antigen (PCNA) is a conserved scaffolding protein that interacts with other proteins essential to DNA damage response and intracellular signaling. ATX-101 blocks this interaction and is thought to result in increased cell death through the interruption of DNA damage repair (43, 44). A phase 2 clinical trial investigating ATX-101 monotherapy for advanced LPS and LMS is ongoing (45) (NCT05116683).

Newer approaches to LMS involve directly targeting the cell’s main DNA damage response machinery, which is comprised of ataxia telangiectasia and Rad3-related protein (ATR), ataxia telangiectasia mutated (ATM) protein kinase and the DNA-dependent protein kinase catalytic subunit (DNA-PKcs) (46, 47). As mentioned, molecular profiling has uncovered recurrent alterations in telomere maintenance genes such as *ATRX* and homologous recombination DNA repair genes (13, 16, 17). Mutations in these pathways may lead to increased dependency on the cell’s DNA damage response machinery for survival. This has led to appealing anti-cancer targets, including ATR inhibitors (ATRi) and DNA-PK inhibitors (DNA-PKi). The ATRi BAY1895344 was tested *in vivo* in uterine LMS sarcoma mouse models harboring *ATRX* mutations. Treatment with BAY1895344 demonstrated growth inhibition compared to vehicle control, with no significant toxicity (47). Two DNA-PKi were also tested, peposertib and AZD7648 in LMS sarcoma models. Co-treatment with low-dose doxorubicin sensitized LMS cells to peposertib or AZD7648 with significant inhibition of LMS cell viability and proliferation. Furthermore, co-treatment of LMS patient-derived xenografts with peposertib and low dose anthracycline significantly inhibited tumor growth in 5 out of 7 models without toxicity. These responses correlated with HR deficiency and *ATRX* inactivation (48). Given these promising preclinical data, ATRi are now being studied in patients with sarcoma. A phase 2 study of ATRi berzosertib in combination with gemcitabine for patients with STS is ongoing (NCT04807816).

### 4.2 Immunotherapy

Immunotherapy has evolved over the past few decades, with tremendous advances in various cancers. Due to these successes, there has been interest in using immunotherapy for the treatment of sarcoma. Several studies have been performed to better understand the tumor immune microenvironment (IME) within sarcoma and translate these findings into novel therapeutic approaches. Dr. Pollack and colleagues investigated the tumor IME in sarcoma for which immunophenotyping of 19 LMS tumors demonstrated a relatively inflamed tumor IME as compared other sarcoma subtypes. For LMS (and undifferentiated pleomorphic sarcoma), there was a higher expression of genes related to antigen presentation and T-cell mediated immunity compared with other subtypes including synovial sarcoma and myxoid/round cell LPS (49). Further investigation within LMS revealed greater immune cell infiltration in soft tissue LMS versus uterine LMS, with soft tissue LMS demonstrating over 2-fold increase in CD8 T-cell and B-cell abundance (50).

Despite the potential for immunotherapy in LMS, clinical trials with immune checkpoint blockade have been disappointing. In the phase 2 trial of pembrolizumab in advanced sarcoma (SARC028), 86 patients were treated, including 10 patients with LMS. There were no objective responses within the LMS population (51). In the phase 2 trial of single agent nivolumab for advanced uterine LMS, none of the 12 treated patients had an objective response and the median PFS was 1.8 months (52). In the phase 2 trials of nivolumab with or without ipilimumab treatment for metastatic sarcoma (Alliance A091401), 43 patients were treated with nivolumab monotherapy, including 15 patients with LMS, and 42 patients were treated with nivolumab plus ipilimumab, including 14 patients with LMS. One of fifteen LMS patients treated with nivolumab monotherapy and two of fourteen LMS patients treated with nivolumab plus ipilimumab demonstrated an objective response, suggesting limited activity in LMS (53).

To potentiate the effects of immune checkpoint blockade, combination approaches with other anti-neoplastic agents such as chemotherapy have been investigated. In a phase 1 trial of gemcitabine and pembrolizumab in LMS and undifferentiated pleomorphic sarcoma, 11/13 treated patients had LMS. There was 1 DLT observed with gemcitabine at 1000mg/m^2^. The maximum tolerated dose was not reached and recommended gemcitabine dose was 1200mg/m^2^ on day 1 and 8 with pembrolizumab 200mg on day 1, for 21-day cycles. Median PFS was 5.1 months and best response at 9 weeks for LMS was stable disease in 8/11 patients. The final results of the dose expansion cohort are pending (54). In a phase 2 trial of eribulin plus pembrolizumab in patients with metastatic STS, 19 patients with LMS were treated and 11/19 had uterine LMS. The PFS rate at 12 weeks was 42.1% which failed to meet the primary endpoint of 60%. The ORR in the LMS population was 5.3% (55). In a phase 2 trial of pembrolizumab in combination with doxorubicin in patients with anthracycline-naïve advanced STS, 30 patients were enrolled including 10 patients with LMS. The median PFS was 5.7 months for all STS. In the LMS population, 4/10 patients (40%) experienced a partial response, demonstrating encouraging activity of this regimen (56). In a phase 1/2 study of ipilimumab, nivolumab, and trabectedin for advanced soft tissue sarcoma, analysis of phase 2 which included 88 evaluable patients with previously untreated STS demonstrated an ORR of 21.6% with 8 complete responses and 11 partial responses. The median PFS was 7 months and median OS was 14 months (57). In LMS-specific subgroup analysis of this trial which included 19 evaluable patients in phase 2, the ORR was 31.6% with 2 complete responses and 4 partial responses. The median PFS was 7.4 months and median OS was 36.1 months (58). The phase 1 results of a phase 1/2 trial of retifanlimab with gemcitabine plus docetaxel for STS (NCT04577014) were recently presented at ASCO 2022. This study included a safety run-in followed by a 3 + 3 dose de-escalation design. Gemcitabine (900mg/m^2^) was administered on days 1 and 8, and docetaxel 75mg/m^2^ on day 8, in 21-day cycles. Retifanlimab (210mg IV flat dose in the run-in portion, and 375mg in the dose de-escalation portion) was administered on day 1 of each cycle starting in cycle 2, and continued as monotherapy after 6 cycles of gemcitabine and docetaxel. Results demonstrated safety and tolerability of this regimen, with the RP2D determined to be retifanlimab at 375mg plus gemcitabine and docetaxel. For the run-in and de-escalation cohorts respectively, ORR was 17% and 50%, disease control rates were 100% and 83%, and PFS rates at 24 weeks were 60% and 44%. Phase 2 is ongoing (59). Other active studies testing immune checkpoint blockade in combination with chemotherapy include a phase 2 study of avelumab with gemcitabine, results are pending (NCT03536780).

Other combination approaches with immune checkpoint blockade have been investigated in sarcoma. Synergistic effects have been observed with the combination of immune checkpoint blockade and antiangiogenic agents in other cancers (60). As a result, this approach is of interest in sarcoma. In a phase 2 trial of pembrolizumab with axitinib (a small molecule tyrosine kinase inhibitor active on VEGF receptors) 33 patients were treated including 6 patients with LMS (uterine LMS = 4, non-uterine LMS = 2). Only 1 patient with non-uterine LMS achieved a partial response (61). An ongoing phase 2 trial is testing the combination of cabozantinib (small molecule inhibitor of receptor tyrosine kinases, VEGF, MET, and AXL) with ipilimumab and nivolumab, and is currently enrolling patients (NCT04551430) (62). Another combination approach involves immune checkpoint blockade with PARPi. PARPi may induce DNA damage and enhance the neoantigen burden thereby potentiating the effects of immune checkpoint blockade. This is being studied in a phase 2 trial of rucaparib and nivolumab for LMS (NCT04624178) for which interim results were presented at CTOS 2022. 20 patients were enrolled, for which 75% had uterine LMS, with a median of 2 prior lines of therapy. Based on 17 evaluable patients, median PFS was 7.8 weeks, OS was 9.4 months. There has been 1 partial response in a patient with uterine LMS with a *BRCA2* mutation. 8 (47%) of patients have had a best response of stable disease (63).

LMS is enriched with tumor-associated macrophages compared to other STS subtypes, which may also provide implications for novel targeted therapies. Macrophages are recruited to tumor sites and can interact with neoplastic cells through the release of various growth factors and cytokines, which may promote tumor angiogenesis, invasion, and metastasis. An increased density of tumor-associated macrophages was associated with worse disease-specific survival in LMS (64, 65). It has also been demonstrated that colony-stimulating factor-1 (CSF1) is a major attractant for macrophages expressed by LMS cells. The expression of genes involved in CSF1 signaling was also associated with worse outcomes in both uterine and non-uterine LMS. As a result, strategies have been aimed to deplete tumor-associated macrophages and inhibit CSF1 signaling in sarcoma. In a phase 1b study of avelumab plus DCC-3014 (inhibitor of CSF1 receptor) in patients with advanced sarcoma, 13 patients were treated including 7 patients with LMS. The combination was deemed to be safe and well-tolerated. Study expansion at the recommended phase 2 dose is ongoing (NCT04242238) (66).

Novel immunotherapy agents are also being tested in LMS. CD40 is a master regulator of immunity which mobilizes multiple arms of the immune system to initiate CD8+ T-cell mediated responses against foreign pathogens and tumors. APX005M is a CD40 agonist that is expected to induce an effective anti-tumor immune response in patients with sarcoma (67). A phase 2 trial of APX005M in combination with doxorubicin in STS is actively recruiting patients (NCT03719430). Another targeted approach involves CD47, a widely expressed transmembrane protein which interacts with signal regulatory protein-alpha on the surface of macrophages to protect tumor cells from phagocytosis. CD47 expression is higher in LMS compared with leiomyoma or normal muscle cells (68). In preclinical models of LMS, an anti-CD47 monoclonal antibody demonstrates increased phagocytic activity of LMS cells, thus inhibiting tumor growth and metastatic spread (68). Consequently, a phase 2 trial testing the CD47 inhibitor (TTI-621) is being studied in combination with doxorubicin for patients with LMS (NCT04996004).

### 4.3 Targeting receptor tyrosine kinases and intracellular signaling pathways

LMS displays substantial mutational heterogeneity and lacks recurrent targetable alterations, including mutations in receptor tyrosine kinases. There are rare circumstances in which actionable gene alterations may be seen in LMS, such as in *ALK*, *FGFR1*, and *NTRK* (17). However, in general due to the lack of targetable mutations, most trials have investigated broadly acting tyrosine kinase inhibitors (TKI) for LMS. As noted earlier, the small-molecule TKI pazopanib is an approved treatment for patients advanced STS who have previously received chemotherapy. However, efficacy in LMS is modest (LMS: ORR = 6%, mPFS = 4.6 months; uterine LMS: ORR 11%, mPFS = 3 months) (8, 9), and there have been ongoing efforts to improve outcomes with other TKIs/TKI combinations.

Clinical trial data examining TKIs in LMS are mixed. Bevacizumab is a monoclonal antibody against VEGF. VEGF normally binds to VEGF receptors, which are family members of receptor tyrosine kinases involved in angiogenesis (69). A phase 3 trial examining the addition of bevacizumab to first-line gemcitabine and docetaxel failed to show improvement in PFS, OS, and ORR (70). Lenvatinib is a small molecule inhibitor that targets fibroblast growth factor receptors (FGFR), PDGFRα, RET, and KIT, in addition to VEGF (71). In a phase 1b/2 study of lenvatinib plus erubulin in advanced LPS and LMS, the phase 1b portion determined the RP2D to be lenvatinib 14mg/day and eribulin 1.1mg/m^2^ on day 1 and day 8 for 21-day cycles. A total of 30 patients were enrolled, including 21 patients with LMS. For the LMS population, the median PFS was 8.6 months with ORR of 19% (4/21, 3 uterine and 1 nonuterine LMS) (72). These data may suggest that the addition of lenvatinib potentiates the effects of eribulin, as historical controls of eribulin monotherapy in LMS exhibit worse outcomes, with a median PFS of 2.2 months, OS of 12.7 months, and ORR of 5%, summarized in **Table 2** (11). Collectively, these data demonstrate promising efficacy for the treatment of advanced LMS.

Cabozantinib is small molecule inhibitor of tyrosine kinases c-MET and VEGFR2, as well as AXL and RET (73). A phase 2 study testing cabozantinib plus temozolomide for advanced LMS is ongoing (NCT04200443). In a small study performed by Dr. Ikeda and colleagues, the addition of bevacizumab to the regimen of cabozantinib and temozolomide for patients with heavily pre-treated uterine LMS demonstrated improved clinical benefit rate (74). Anlotinib is a multi-target TKI including VEGF1-3, FGFR1-2, PDGFRβ, and KIT (75). A phase 2 trial of anlotinib was tested for first-line treatment in patients with advanced STS, including LMS. Results (all STS) demonstrated a median PFS of 7.1 months, with ORR of 2.7% (76). A randomized phase 3 study of anlotinib versus dacarbazine after failure of prior therapy in several STS subtypes is ongoing, however enrollment in the LMS cohort has been suspended and results are pending (NCT03016819).

Olaratumab is a monoclonal antibody against tyrosine kinase PDGFRα, blocking its interaction with PDGF. A randomized, phase 2 study of doxorubicin plus olaratumab, followed by olaratumab monotherapy in anthracycline-naïve STS demonstrated promising results, with improvement in mPFS and mOS (77). This led to the confirmatory, randomized, phase 3 ANNOUNCE trial of doxorubicin with or without olaratumab in anthracycline-naïve advanced STS, including LMS. For both STS and LMS, there was no significant difference in primary endpoint of mOS between doxorubicin plus olaratumab versus doxorubicin (LMS: 21.6 versus 21.9 months) (78). LMS accounted for a smaller percentage of total subtypes in phase 2 versus phase 3 (36% versus 46.1%), therefore the benefit seen in phase 2 may be weighted towards non-LMS populations (77, 78). Based on these results, olaratumab is not part of standard of care treatments for STS and LMS.

Other approaches to LMS treatment target intracellular pathways involved in tumorigenesis. In LMS, aberrant PI3K/AKT/mTOR signaling has been seen due to *PTEN* loss and amplifications of *IGF1R, AKT, RICTOR*, and *mTOR* (12). Unfortunately, clinical trials targeting this pathway have demonstrated limited activity. In the phase 2 trial of dual mTORC1/mTORC2 inhibitor MLN0128 (sapanisertib), 111 patients were treated, including 76 patients with LMS. For the LMS population, PFS was 2.1 months with ORR of 3% (79). Another intracellular target in LMS includes cyclin-dependent kinase inhibition. CDK4 amplification has been seen in some LMS tumors (80, 81). In preclinical models of LMS, treatment with the CDK4/6 inhibitor palbociclib resulted in decreased cell proliferation and induction of G0/G1 phase cell-cycle arrest (81). Consequently, a phase 2 trial of the CDK4/6 inhibitor ribociclib in combination with mTOR inhibitor everolimus was tested in patients with dedifferentiated LPS and LMS with retained Rb expression. 24 patients with LMS were treated, including 14 with uterine LMS. The primary endpoint was progression free rate at 16 weeks, with treatment declared as promising if at least 8/24 patients were progression free at 16 weeks. Final data on the primary endpoint is pending, however of the 22 patients with complete data, 6/22 (27%) met the primary endpoint and median PFS was 19.6 weeks, with no objective responses (82).

### 4.4 Metabolism

A newer approach to the treatment of sarcoma includes targeting aberrant metabolic processes associated with oncogenesis. In a study of 708 sarcoma tumor samples, argininosuccinate synthase 1 (ASS1) expression was lost in 87%. ASS1 is the rate-limiting enzyme in the conversion of citrulline to arginine in the urea cycle. The loss of ASS1 makes cells dependent on extracellular sources of arginine for survival. As a result, cancer cells lacking ASS1 may have metabolic vulnerabilities (83). Preclinical studies demonstrate synergistic effects with the treatment of arginine depleting enzyme PEGylated arginine deiminase (ADI-PEG20) in combination with gemcitabine and docetaxel. The main transporter of gemcitabine is human equilibrative transporter 1 (hENT1). Priming of tumors with ADI-PEG20 and docetaxel resulted in the stabilization of c-MYC, potentiating the effect of gemcitabine treatment through an increase in hENT1 expression (84).

Given promising preclinical data, a phase 2 study of ADI-PEG20 in combination with gemcitabine and docetaxel for STS was performed by Dr. Van Tine and colleagues. 75 patients who received at least one prior line of therapy were treated. The trial underwent two dose reductions due to prolonged neutropenia and thrombocytopenia: gemcitabine was reduced from 900mg/m^2^ to 750mg/m^2^, and again to 600mg/m^2^. Docetaxel was reduced from 75mg/m^2^ to 60mg/m^2^. For those receiving gemcitabine 600mg/m^2^ + docetaxel 60mg/m^2^, PFS and OS were 7.2 and 22.5 months, respectively for the LMS group. 8% of patients (6/75) achieved a complete response, including 3 of the 6 with LMS (85). A phase 3 randomized trial of ADI-PEG20 with gemcitabine plus docetaxel is planned.

### 4.5 Novel chemotherapy combinations

Currently approved chemotherapy regimens for LMS demonstrate modest efficacy therefore studies have investigated novel chemotherapy combinations in order to improve benefit. Preclinical data demonstrated promising activity with trabectedin and doxorubicin (86, 87). As a result, this combination was studied in two phase 1 studies, which confirmed safety and tolerability when used with granulocyte colony-stimulating factor (88, 89). This led to a phase 3 trial of doxorubicin plus trabectedin versus doxorubicin as first-line treatment for patients with advanced LMS. Patients were randomly assigned (1:1) to receive doxorubicin alone (75 mg/m^2^) once every 3 weeks for up to six cycles versus intravenous doxorubicin (60 mg/m^2^) plus intravenous trabectedin (1.1 mg/m^2^) once every 3 weeks for up to six cycles followed by maintenance with trabectedin alone. The median PFS was significantly longer with doxorubicin plus trabectedin versus doxorubicin alone (12.2 months vs. 6.2 months), at the expense of higher toxicity with grade 3-4 adverse events reported in 52% of patients in the doxorubicin group alone versus 96% in the doxorubicin plus trabectedin group (90).

Another promising novel chemotherapy combination in LMS includes unesbulin (PTC596) plus dacarbazine. Unesbulin is an investigational small-molecule tubulin binding agent. In preclinical LMS models, unesbulin was shown to potentiate the activity of dacarbazine (91). As a result, this was developed into a phase 1b study of unesbulin plus dacarbazine for the treatment of patients with advanced LMS. Results were presented at both ASCO 2022 (92) and CTOS 2022 (93). The RP2D of unesbulin was determined to be 300 mg orally BIW with dacarbazine 1,000 mg/m^2^ IV every 21 days. As of the most recent presentation of data at CTOS 2022, there were 33 evaluable patients, 14 with nonuterine LMS and 19 with uterine LMS. Median prior lines of therapy were 3. The ORR was 18.2% with disease control rate of 51.5% at 12 weeks (93). A randomized, placebo-controlled phase 2/3 trial has been developed and is actively recruiting patients (NCT05269355).

## 5 Discussion

LMS is a rare and aggressive cancer that displays significant clinical and biologic heterogeneity. As a result, LMS is challenging to treat in the advanced setting. Our understanding of LMS pathophysiology has progressed through the use of molecular profiling resulting in the development of novel and targeted treatment approaches. There are several approaches that appear promising thus far. These include targeting DNA damage repair pathways with olaparib and temozolomide, combination chemotherapy with unesbulin plus dacarbazine, several new immunotherapy targets such as CD40 or CSF1 receptor, novel immunotherapy combinations with chemotherapy such as with doxorubicin or with targeted drugs such as cabozantinib, and exploitation of metabolic vulnerabilities using ADI-PEG20 with gemcitabine plus docetaxel. Some of these regimens are now being investigated or will soon be investigated in larger randomized phase 3 clinical trials and have the potential to improve current standards of care in advanced LMS.

The future of LMS treatment is contingent upon a greater understanding of tumor biology and continued development of prospective clinical trials based on molecular findings. A challenge in studying LMS is how to account for the heterogeneity of this disease, especially in the context of a clinical trial. As we have seen, various multiomic molecular profiling studies have identified subtypes within LMS that display vastly different clinical outcomes and are not necessarily related to anatomical site. Despite this, LMS enrollment onto clinical trials continues as a homogenous entity. There may be clinically meaningful effects of a study drug for certain LMS populations that are not obviously apparent based on overall results, potentially leading to missed therapeutic benefit. This may be the case for several of the negative trials presented in this review, including larger negative phase 3 studies such as ANNOUNCE (78) as mentioned above, EORTC 62012: doxorubicin alone versus combination with ifosfamide (94), PICASSO III: doxorubicin alone versus combination with palifosfamide (95), and TH CR-406/SARC021: doxorubicin alone versus combination with evofosfamide (96). Future enrollment and treatment selection based on molecular data may ultimately reveal a preferential response for an LMS subtype that would not otherwise be identified.

Another challenge in treating LMS is that its most common molecular alterations involve loss of tumor suppressor function in *RB*, *TP53* and *PTEN* (16, 17), which are not currently actionable using existing cancer therapeutics. Furthermore, PD-1 inhibition has not proven efficacious in LMS. New insights into the immunosuppressive features of the LMS tumor IME are needed to identify novel targets for immunotherapy-based approaches. As we have seen, response to immune therapy in LMS is very infrequent and this speaks to the need for biomarker development for this and for other sarcoma subtypes. Tertiary lymphoid infiltrates have been suggested as a biomarker for immunotheapy in sarcomas but this has yet to be fully evaluated prospectively (97).

Future trials should continue to investigate the molecular evolution of LMS, treatment effects on pathology, and discovery of potential biomarkers. Successful translation of molecular findings in LMS will require ongoing preclinical modeling, thoughtful clinical trial design, strong academic collaborations, and prospective correlative analysis. These considerations are necessary for the current and future development of novel therapeutic agents that will improve clinical outcomes for patients with advanced LMS.

## Author contributions

KL drafted the manuscript. MI, SB, and GS helped to revise the manuscript. All authors contributed to the article and approved the submitted version.
